# Genome Re-Sequencing of Semi-Wild Soybean Reveals a Complex *Soja* Population Structure and Deep Introgression

**DOI:** 10.1371/journal.pone.0108479

**Published:** 2014-09-29

**Authors:** Jie Qiu, Yu Wang, Sanling Wu, Ying-Ying Wang, Chu-Yu Ye, Xuefei Bai, Zefeng Li, Chenghai Yan, Weidi Wang, Ziqiang Wang, Qingyao Shu, Jiahua Xie, Suk-Ha Lee, Longjiang Fan

**Affiliations:** 1 Department of Agronomy & James D. Watson Institute of Genome Sciences, Zhejiang University, Hangzhou, China; 2 Institute of Nuclear Agricultural Science, Zhejiang University, Hangzhou, China; 3 Department of Pharmaceutical Sciences, North Carolina Central University, Durham, North Carolina, United States of America; 4 Department of Plant Science and Research Institute for Agriculture and Life Sciences, Seoul National University, Seoul, Korea; University of Illinois, United States of America

## Abstract

Semi-wild soybean is a unique type of soybean that retains both wild and domesticated characteristics, which provides an important intermediate type for understanding the evolution of the subgenus *Soja* population in the *Glycine* genus. In this study, a semi-wild soybean line (Maliaodou) and a wild line (Lanxi 1) collected from the lower Yangtze regions were deeply sequenced while nine other semi-wild lines were sequenced to a 3-fold genome coverage. Sequence analysis revealed that (1) no independent phylogenetic branch covering all 10 semi-wild lines was observed in the *Soja* phylogenetic tree; (2) besides two distinct subpopulations of wild and cultivated soybean in the *Soja* population structure, all semi-wild lines were mixed with some wild lines into a subpopulation rather than an independent one or an intermediate transition type of soybean domestication; (3) high heterozygous rates (0.19–0.49) were observed in several semi-wild lines; and (4) over 100 putative selective regions were identified by selective sweep analysis, including those related to the development of seed size. Our results suggested a hybridization origin for the semi-wild soybean, which makes a complex *Soja* population structure.

## Introduction

**Figure 1:**
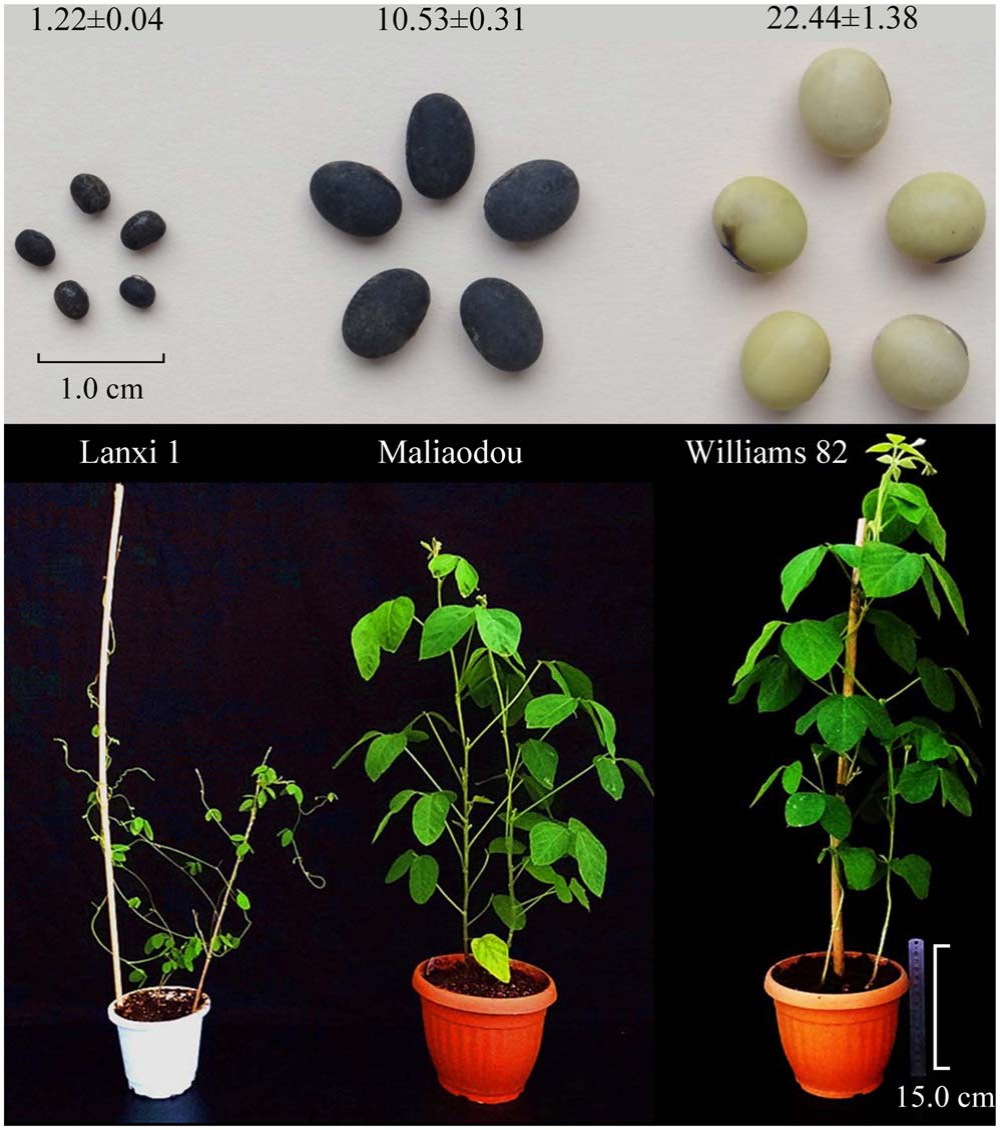
Phenotypes of cultivated, semi-wild and wild soybeans used in the present study. Seed weight (per 100 seeds) and plant architecture are shown.

The genus *Glycine* has two subgenera: *Glycine* and *Soja*. The latter one consists of the cultivated soybean (*Glycine max*) and its progenitor wild soybean (*G. soja*). *G. max* is an important cash crop for dietary protein and oil world-wide. It is generally believed that *G. max* was domesticated from its annual wild relative *G. soja* in China around 5,000–6,000 years ago [1], [2]. After domestication, *G. max* displays distinct differences in several traits from *G. soja*. For example, *G. soja* has much smaller seeds (<3.0 g per 100 seeds) and a darker seed coat, whereas *G. max* (generally >9.0 g per 100 seeds) has a yellow seed coat [3]. Besides the above two species with distinct morphological characters in the subgenus *Soja*, an intermediate type can be found in accessions of landrace soybean germplasm collections or wild line collections in China [4]–[6]. For example, an intermediate type known as *G. gracilis* has been described as a semi-wild soybean, which usually has an intermediate seed weight (>3.0 g per 100 seeds) with a dark seed coat [6], [7]. In China, this form is also a popular type of soybean cultivated in the northeast and the Yangtze regions, because it usually has a very high seed germination rate and a short growth period as well as a robust adaptability to various environments [4], [8]. For example, Maliaodou (refers to “beans for horse fodder” in Chinese) is a popular growing semi-wild soybean in the Yangtze regions. In the modern Chinese Soybean Breeds [9], Maliaodou (No. 495) was listed as a landrace in the Jinhua, Zhejiang province. It has a dark seed coat with a mud film, which in general is a typical phenotype for wild species (Fig. 1). However, it possesses an erected plant architecture with a main stem and large leaves, which are similar to cultivated soybean lines. In brief, the intermediate type, i.e. semi-wild soybean, provides an important population for the subgenus *Soja* and may be beneficial in shedding light on its origin and the process of soybean domestication.

As a new species, *G. gracilis* was first proposed by Skvortzow [10]. However, its origin has been a subject of intense debate. Some early studies denied its novelty as a new species and proposed that it be incorporated into *G. max* [11]. Two original views have been projected for *G. gracilis*: an intermediate evolutionary type between *G. soja* and *G. max* [12] and a hybridization origin from *G. max* and *G. soja* [13]. The latter hypothesis was supported by several studies based on the analyses of the frequency/distribution of alleles [14] and molecular markers [15]–[17]. A recent observation of inter-species gene flow, which captured the natural occurrence of introgression between cultivated and wild accessions, provided further phenotypic evidence to support the hybridization hypothesis [6], [18], [19]. However, the origin of this semi-wild type of soybean has not clearly been established.

Gene flow between *G. max* and the wild relative *G. soja* has been observed [7], [19], [20]. Despite significant phenotypic differences between the two species, no reproductive isolation has been found yet. Introgressions between wild and cultivated soybeans were revealed by RFLP [15] and SSR [3], [6], [17], [19], [21]–[23]. It has been proven that the introgression between wild and cultivated soybean is bidirectional rather than unidirectional, i.e. from wild to cultivated soybean populations [21], [24], [25], and also cultivated soybean into the wild population [18], [19].

The *Soja* population has been investigated using diverse molecular markers such as SSR [3], [6], [17], [19], [21]–[23]. Based on 111 fragments from 102 soybean genes, the genetic bottleneck associated with artificial selection in soybean was first illustrated [26]. Later, SNPs based on genome-resequencing [25], [27], [28] or SNP chips [23], [29] were identified in the wild and cultivated soybeans, and this provided the first investigation of population structure and the estimation of loci under domestication and genetic improvement from the whole soybean genome. With the low cost and high-throughput sequencing platforms, genome re-sequencing of a representative set of semi-wild soybean accessions is now possible, therefore providing us with a unique opportunity to investigate a more comprehensive subgenus *Soja* population structure and the origin of the semi-wild soybean at the genomic level.

In this study, we sequenced 10 semi-wild soybeans with a wild line and then analyzed our sequence data together with other available genomic data from wild and cultivated soybean lines. Our results demonstrate the hybridization origin of semi-wild soybean and a high rate of genetic introgression among the members of the subgenus *Soja*, which resulted in a mixed population structure including wild, semi-wild and cultivated soybean.

## Materials and Methods

### Plant materials

The semi-wild soybean Maliaodou and wild soybean (*G. soja*) Lanxi 1 were collected from the Jinhua basin in Zhejiang province, China (N29°04′, E119°38′). Other semi-wild soybeans were collected from wide geographical locations in China and kindly provided by Institute of Crop Sciences, Chinese Academy of Agricultural Sciences (CAAS) (Table S1). No specific permissions were required for the location, and the study did not involve endangered or protected species.

### Genomic sequencing

Green leaves from a single plant of each accession were used for DNA extraction following previously described protocol [30]. Two libraries with 500 bp and 2 kb insertion sizes for Lanxi 1 and one library with a 500 bp insertion size for each of other lines were generated for Illumina Hiseq2000 sequencing platform. Paired-end (PE) reads with 100 bp were determined and a clean data set was collected from raw reads, which were pre-processed to remove adaptors to filter out low quality reads (≥50% of its nucleotides with <Q20). E-corrections were performed with the program “Correction” to reduce the low frequent K-mer for better assembly [31].

### Genome assembly

To get the draft genome sequences of Maliaodou and Lanxi1, different K-mer sizes ranging from 29-mer to 55-mer were tried to perform *de* *novo* assembly by SOAPdenovo v1.05 with clean paired-end reads [31]. The best assembly draft data (i.e., its contigs with the longest N50) was achieved at the 49-mer parameter for both Maliaodou and Lanxi1. Scaffold construction was performed based on the paired-end information of reads, and the gaps between the scaffolds were then closed by GapCloser v1.12 [31].

For the assembly of the Lanxi 1 chloroplast genome, the clean paired-end reads of Lanxi 1 that could be mapped to the known *G. max* chloroplast genome (NC_007942 [32]) by Bowtie2 v2.0.5 [33] were collected. The average mapping depth reached above 4000×, which is ∼80 times of the whole genome mapping depth. Collected reads with low mapping depth (<250x) was filtered in our assembly effort. The remaining reads were used for *de* *novo* assembly using Velvet v1.2.07 [34] with 51-mer length. Considering the possibility that some reads from the chloroplast genome may be missed due to the used 250× threshold, we closed the gaps using all the clean reads by the software GapCloser v1.10 [35]. The overlap-based CAP3 [36] was utilized to merge redundant sequences for assembly refinement.

### Genome annotation

Repeat regions in the assembled genome of Lanxi 1 were first identified using *de* *novo* methods implemented in RepeatScout v1.0.5 [37] and further masked by homology-based RepeatMasker v3.3.0 [38]. Genes were predicted using Augustus v2.5.5 [39] with Arabidopsis as a species parameter. Functions of the predicted genes were annotated by the BLASTP search against nr [40] and the annotated gene set of Williams 82 genome (ftp://ftp.jgi-psf.org/pub/compgen/phytozome/v8.0/Gmax_v1.0/annotation/Gmax_109_peptide.fa.gz) [41] with e-value<1e-5 as a threshold value.

### SNP calling and structural variations

The clean reads of each soybean line were aligned to the Williams 82 reference genome (ftp://ftp.jgi-psf.org/pub/compgen/phytozome/v8.0/Gmax_v1.0/assembly/Gmax_109.fa.gz) using Bowtie2 v2.0.5 with default settings [33]. Consecutive steps were applied for SNPs detection. Samtools v0.1.18 [42] was used for SNP calling with the parameter –C = 50, which aims to reduce the effect of reads with excessive mismatches. In order to avoid paralogue inference, –q = 1 as threshold was used to filter reads that could align to multiple regions. Besides, to alleviate the false positive calling result due to relative low sequencing depth of some semi-wild soybean lines, we combined the variant calling information of all the 43 soybean accessions (31 by Lam *et al*. [27], one by Kim *et al*. [46] and 11 by this study), and selected SNPs present in at least two accessions or present in only one accession but with above 10 reads supported. The bam file produced from the mapping procedure was further analyzed for structural variations detection by BreakDancer v1.1 [43] with default parameters. Structural variations were displayed using Circos v0.62 [44].

### Genetic diversity estimation

The average pairwise divergence (*π*, [45]) within a population was estimated for the wild, semi-wild and cultivated soybean populations. An in-house custom PERL script was applied for the estimation. Based on SNP calling results of each line, all variant sites across the whole genome were identified for each population and the number of nucleotide substitutions per site was estimated. The whole length of the reference genome was taken as a total number of nucleotides for *π* estimation.

### Identification of cultivated and wild soybean-specific sequences

Nineteen wild lines (17 by Lam *et al*. [27] and two by Kim *et al*. [46] and this study) and 15 cultivars (14 by Lam *et al*. [27] and one Williams 82 by Schmutz *et al*. [41]) were used to identify the cultivated and wild soybean-specific sequences. Any sequences in the reference genome Williams 82 (ftp://ftp.jgi-psf.org/pub/compgen/phytozome/v8.0/Gmax_v1.0/assembly/Gmax_109.fa.gz), which could not be mapped by any reads from Lanxi 1 and other wild lines, were identified and defined as cultivated-specific sequences. For wild-specific sequences, the reads from the wild line Lanxi 1, which were not mapped into Williams 82, were collected and assembled by *De novo* into a 10 Mb set using Velvet v1.2.07 [34] with optimal K-mer size of 43 bp. The set was further mapped by the reads from the other 14 cultivars (Lam *et al*. [27]). Those sequences that could not be mapped by any reads from the 14 cultivated lines were considered as wild-specific sequences. Moreover, for identification of wild/cultivated common sequences, the genomic regions mapped by the all 14 cultivated lines but were not wild Lanxi 1 were considered as to be common sequences of cultivated soybeans, while the common-mapped regions in the *de* *novo* assembly of Lanxi 1 (as a wild reference genome) by the 18 wild lines, but not the Williams 82, were selected as common sequences of wild soybeans. In the process of identification specific or common regions, BWA v0.6.1 [54] was applied for mapping with default parameters. Mapping coverage and location information was obtained from the Samtools v0.1.18 using ‘Samtools depth’ parameter. The unmapped regions with at least 100 bp in length were extracted by in-house PERL scripts.

### Phylogenetic tree and population structure

To collect a solid SNP dataset for constructing a *Soja* phylogenetic tree, the loci with a minimum coverage of three bases for each line across the genome were first selected, and a total number of 3,794,973 loci were found in all soybean lines. Heterozygous SNPs and the adjacent SNPs within 50 bp detected in each individual were further excluded for further genotyping. Finally, a total of 7,424 SNPs were used to construct the phylogenetic tree and population structure. The neighbor-joining tree was constructed by MEGA5.2 [47] with bootstrap support (1,000 replicates). The program STRUCTURE v2.3.4 [48] was applied for population structure; and the length of burn-in period was set to 100,000 with 100,000 MCMC reps afterwards. The number of genetic clusters was assigned using the Delta*K*-method described by Evanno *et al*. [49]. The number of clusters (*K*) was tested ranging from 2 to 9 with 8 replicates per *K*.

### Selection analysis

To detect signals of recent selection, a method based on reduced pooled heterozygosity [50], [51] was used. Considering the unbalanced sequencing data, in order to alleviate bias we preprocessed the sequences from each line by randomly choosing the fastq sequences to make all lines with 3∼4× sequencing depth. Three subgroup pools (wild, semi-wild and cultivated) with pretreated sequence data from each line were created. By taking the genome of Williams 82 as a reference, variable sites in each pool were identified with the coverage ranging from 10 to 500. Reference and variant allele counts (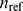 and 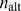) at identified SNP positions from each pool were used to identify selections in 100 kb sliding windows with a step size of 50 kb. For a 100 kb window along the reference genome, the pooled heterozygous (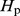) was calculated by the formula: 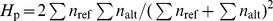, where 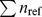 and 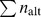 are the sums of 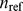 and 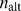 for all SNPs in the window. *Z* transformation (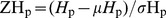) was then applied to locate the putatively selected regions from the extreme tails by a threshold of 4 standard deviations as previously used [50], [51].

### GO enrichment analysis

The genes located in the selected regions were extracted from the soybean gene annotation file in Phytozome (ftp://ftp.jgi-psf.org/pub/compgen/phytozome/v8.0/Gmax_v1.0/annotation/Gmax_109_gene.gff3.gz), and the GO enrichment study conducted through AgriGO and ‘*Glycine max*’ was set as the species setting (http://bioinfo.cau.edu.cn/agriGO/) [52]. The p-value and FDR criteria for the considered enrichment GO terms were <0.0001 and <0.05, respectively.

### Experimental validation

To confirm the genomic variations in Maliaodou, 25 variations (10 SNPs and 15 indels) were selected for experimental validation. Primers were designed based on the flanking sequences of the variation sites using the on-line software Primer3 (http://frodo.wi.mit.edu/) (Table S2). The PCR reaction was carried out as described previously [53]. The PCR amplification products were checked by electrophoresis on 1.0% (w/v) agarose, then purified and sequenced directly or through clone sequencing using the Sanger platform (ABI 3737XL) (Sunny, Shanghai). In the clone sequencing effort, at least five clones were sequenced for each sample.

## Results

### Genome re-sequencing

Most of the semi-wild soybeans possess an erected plant architecture with a main stem and/or large leaves, which are similar to cultivated soybean lines (Maliaodou is shown in Fig. 1). The ten semi-wild soybeans have an intermediate seed size (4.34–12.32 g), which is between the wild line (e.g. Lanxi 1, 1.22±0.04 g) and the cultivated line (e.g. Williams 82, 22.44±1.28 g) (Fig. 1; Table S1).

All semi-wild soybeans were sequenced into a ∼3-fold genome coverage by high-throughput sequencing; Maliaodou was further sequenced into a 41-fold coverage (Table S3). In order to obtain a reference for the wild soybean genome for comparative analysis, a wild line Lanxi 1 was collected from the same location as Maliaodou and was sequenced into a 55-fold genome coverage by two libraries with different insertion sizes. After *de* *novo* assembly, a 929.9 Mb genome with contig/scaffold N50 sizes of 21.7/51.0 kb was obtained and used as a wild-type reference in the following studies. A total of 56,298 genes were further annotated, of which 48,240 were able to find their orthologs (hits) in the cultivated reference genome Williams 82 (Table S3). For the assembly of the Lanxi 1 chloroplast genome, we finally obtained complete chloroplast genome of Lanxi 1 with length of 152,199 bp, which harbors 139 protein coding genes. The assembly information is available in GenBank under the accession number KC779227.

### Genetic diversity

**Figure 2:**
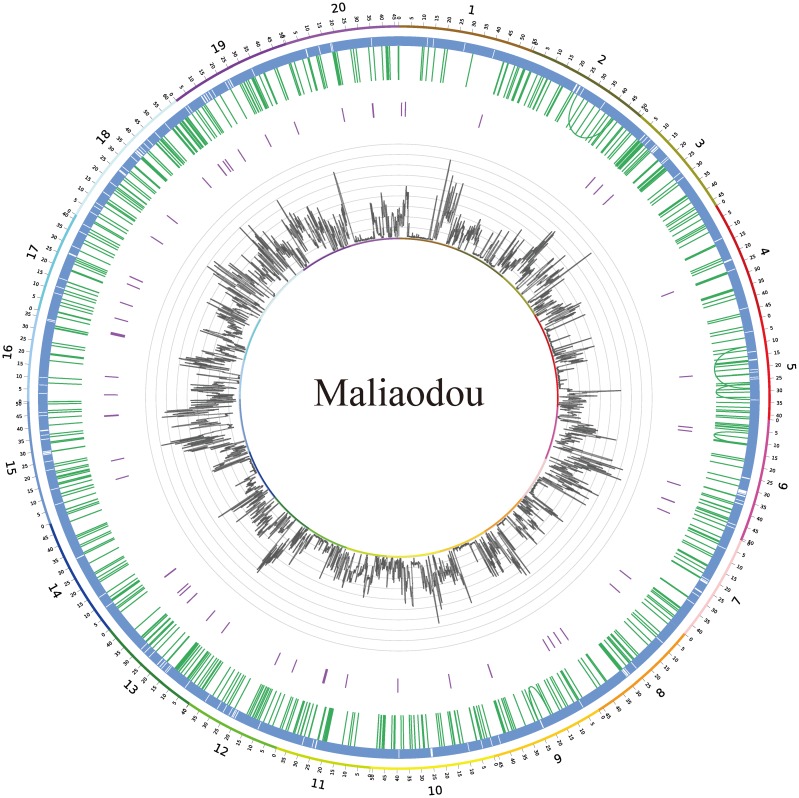
Genomic variations between the semi-wild genome Maliaodou and the reference genome Williams 82. Circles from outside: 20 chromosomes labeled with different colors; in the blue bars, each white vertical line presents a >10 kb un-mapped region in the reference genome; green and purple vertical lines present the intra-chromosomal translocations and inversion events, respectively. Distribution of the SNP density of the Maliaodou is labeled with grey lines.

Variation calling for 43 soybean accessions was carried out using the Williams 82 genome as a reference (Table S4ab). After SNP calling by Samtools v0.1.18 [42] and further steps to alleviate false positive calling by combining the variant calling information of all 43 soybean accessions, a total number of 7,704,637 SNPs were identified. Summary of shared (supported by at least two accessions) and unique variations (supported by at least 10 reads) of each accession is listed in Table S4a. In general, relatively lower variations were observed in the semi-wild soybeans than in the wild lines. The average variation of semi-wild lines had 910,373 SNPs and 38,258/32,907 insertion/deletions. Based on the available genomic data of 19 wild soybean genomes from previous studies [27], [46] and the current study (Lanxi 1), the wild lines had an average of 1,628,253 SNPs and 110,181/94,292 insertion/deletions (Table S4b). For Maliaodou, 1,587,320 SNPs and 111,399/92,112 small insertions/deletions (indels) (<5 nt) were observed compared to the reference (Fig. 2). About 14,157 large regions (>1 kb) or 27.8 Mb cumulated sequences (about 2.8% of the reference genome) in the reference genome were not mapped by any reads of Maliaodou (Table S5a). Among 14,157 regions, 257 were over 10 kb in size (the largest one with 83.5 kb); they are shown in Fig. 2. The results were similar to those obtained by another approach based on information of paired-ends reads using BreakDancer [43], which detected 2,983 deletion events with 8.7 Mb cumulated sequences (score >60). Meanwhile, limited inversion (total number of 60) and translocation (160 and 419 intra- and inter-chromosomal) events were observed in the semi-wild (Table S5b). We also measured genetic divergence of wild, semi-wild and cultivated soybeans using the parameter *π* [45]. The *π* value was 1.416×10^−3^ for semi-wild, which is intermediate between the wild (2.173×10^−3^) and cultivated soybeans (1.332×10^−3^).

Unusual high heterozygous rates (0.19–0.49) were observed in several semi-wild soybeans (Table S4b). For example, a heterozygous rate of 0.49 was observed among the 1,587,320 SNPs in Maliaodou. Using the same pipeline, Lanxi 1 and most other wild lines were found to have lower heterozygous rates (<0.05) except for three lines with 0.12–0.15 (Table S4b). Similar higher heterozygous rates (>0.35) were also observed for indels in Maliaodou but not in any wild lines.

To confirm the variations, especially the unexpected high heterozygosity of Maliaodou, an independent SNP calling a different mapping algorithm implemented in BWA v0.6.1 [54] was carried out for Maliaodou, which is less sensitive but more specific than Bowtie2 v2.0.5 [33]. The results showed 0.50, 0.35, and 0.36 heterozygous rates for SNP, insertion and deletion, respectively (Table S4b). When 25 variations (10 SNPs and 15 indels) were selected for experimental validation by traditional Sanger sequencing, 21 (84%) were confirmed (Table S2). Three indels caused by repetitive sequences were not validated, perhaps due to sequencing errors [55]. The high heterozygous rates were also consistent with our fragmented assembly result of the Maliaodou genome (contig N50 size = 0.5 kb). Previous studies have indicated that the heterozygous rate is one of major disruptive factors in the algorithm for genome assembly [56].

### Phylogenetic relationship and population structure

**Figure 3:**
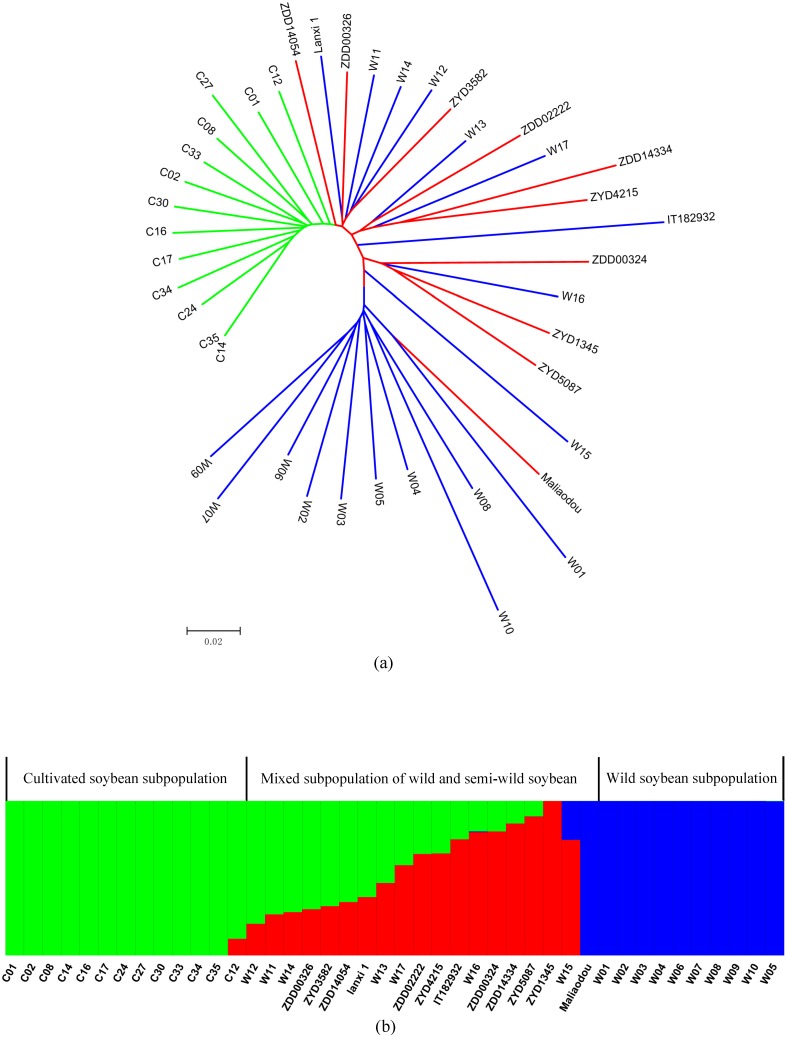
Phylogenetic tree and population structure of wild, semi-wild, and cultivated soybeans. (a) A neighbor-joining phylogenetic tree constructed using SNP data. Cultivated, semi-wild, and wild soybeans are labeled with green, red, and blue lines, respectively. (b) Bayesian clustering (STRUCTURE, *K* = 3) of soybean accessions were grouped based on their species. Cultivated lines were designated with ‘C’ as prefix while wild lines were with ‘W’ as prefix except IT182932 (Korean) and Lanxi 1 (this study). Semi-wild lines were titled with ‘Z’ as prefix except Maliaodou.

A phylogenetic relationship of the 30 re-sequencing wild and cultivated soybean genomes has been analyzed. One of the cultivated soybean accessions (C19) was excluded due to its high admixture [27]. Based on their phylogenetic structure, 10 semi-wild soybean lines from this study and two additional wild soybeans (Lanxi 1 in this study and a line from South Korea by Kim *et al*. [46]) were added into the tree (Fig. 3a). The semi-wild soybeans were not grouped into an independent branch but scattered into wild subgroups.

Seed size is a key target trait of soybean domestication as cultivated soybeans usually have a bigger seed size. According to the phylogenetic tree (Fig. 3a), species with a large seed size (cultivated lines in green) are generally separated from those with a smaller seed size (wild lines in blue). However, in the mixed branch with semi-wild soybeans (in red), their seed weight seems to not be consistent with their evolutionary relationship (Fig. S1). The result indicated that seed size, the most important trait under domestication selection, could hardly be used as a single factor to estimate soybean divergence, especially for those in the intermediate evolutionary stage. This is consistent with the fact that seed size is merely one of the target traits during soybean domestication and is significantly affected by the environmental conditions [57].

Bayesian clustering (STRUCTURE) of soybean accessions was further carried out. Using the method described by Evanno *et al*. [49], Δ*K* reached a peak when *K* was set to 3 (Table S6), indicating that *K* = 3 is optimal for the population structure. When we ordered the accessions based on their genetic background, they could be divided into three subgroups: a cultivated, wild, and mixed subgroup (including both wild and semi-wild soybeans) (Fig. 3b). These results are consistent with our phylogenetic results (Fig. 3a). Similarly, semi-wild soybeans do not have an independent subpopulation and are mainly grouped with wild soybeans.

### Selective sweep analysis

**Figure 4:**
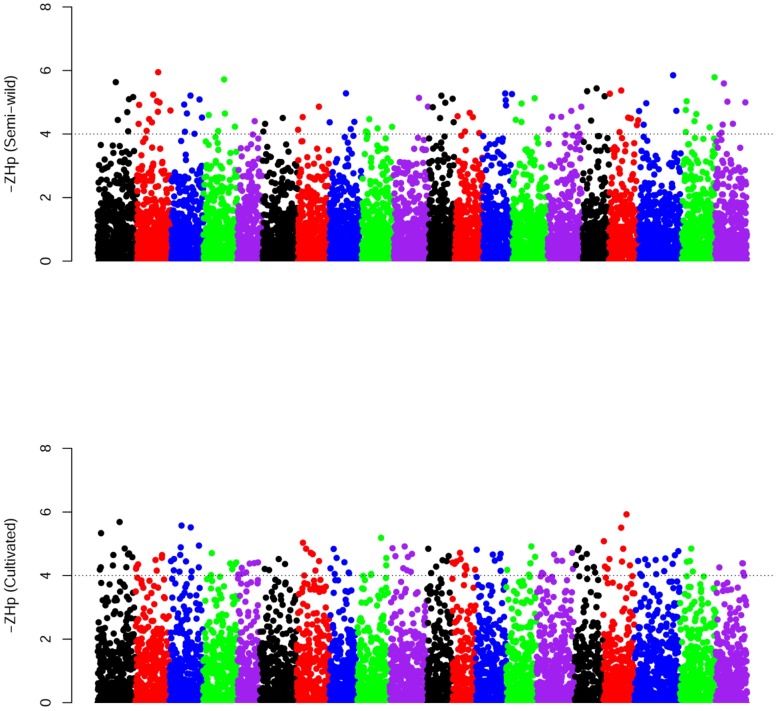
Summary of selective sweep analysis. Distribution of *Z*-transformed average pooled heterozygosity (*H_p_*) in semi-wild (top) and cultivated soybeans (bottom), respectively. The negative end of the *Z*(*H_p_*) distribution plotted along soybean autosomes 1–20 are shown. A dashed horizontal line indicates the cut-off (*Z*<–4) used for extracting outliers.

Crops usually experience two stages (domestication with subsequently genetic improvement) during the evolutionary process from wild-type, and semi-wild is a transitional point between these two stages [58]. To detect signals of artificial selection, we searched the wild, semi-wild, and cultivated soybean genome for selection regions with reduced pooled heterozygosity (*H_p_*). Using autosomal 100 kb as the scanning window suggested by Axelsson *et al.* [51] and Chung *et al*. [28], several regions in cultivated and semi-wild soybean populations had extremely low heterozygosity rates (Fig. 4; Table S7a). By the threshold of at least four standard deviations away from the mean (*Z*(*H_p_*)<–4), a total of 102 and 144 putatively selected regions could be detected in the semi-wild and cultivated populations, respectively. It is reasonable to believe that more selected loci could have been identified in the latter because cultivated population experience additional artificial selection in recent genetic improvements [26].

In the 144 selected loci, only 101 loci were detected in the cultivated soybeans, but not in the wild or semi-wild soybean were *Z*(*H_p_*)<–2 was as a threshold, suggesting that they are the putative target loci under recent genetic improvements. The 101 loci include 282 protein-coding genes (Table S7bc), which could play a vital role in agronomy-related traits. We performed GO enrichment analysis on the 282 genes and identified 9 gene ontology terms with FDR (false discovery rate) <0.05 (Table S8). Interestingly, the GO categories were enriched in embryonic and embryo sac development as well as megagametogenesis, which are involved in the development of seed size. The variants within these genes may lay a genetic foundation to the seed size evolution.

## Discussion

### A mixed semi-wild group complicated population structure of the subgenus *Soja*

It is believed that the cultivated soybean (*G. max*) was domesticated from the wild *G. soja* [1], [2]. Our results demonstrate a complex transitional stage from wild to cultivated soybean: a mixed population including a series of semi-wild types, which suggests no distinct or independent transitional period for soybean domestication. The complex evolutionary situation might be due to the hybridization events between domesticated and wild soybeans, which frequently occurred in natural field conditions (see next section for details). Many crops have had significant reproductive isolation from their wild progenitors after domestication, which made their phylogenetic relationship clearly (such as rice [59]). Our results are consistent with the observation by Wang *et al*. [6] who clearly revealed that there is no existence of a transitional intermediate ancestor between the wild and cultivated soybean. Furthermore, our results do not support the semi-wild soybean as an independent species (*G. gracilis*). Apparently, the mixture of semi-wild and wild accessions in the phylogenetic tree not as an independent sub-branch (Fig. 3a) strongly suggests that the semi-wild soybeans belong to the wild category and should be considered a variant or subclade of *G. soja*. However, in order to fully understand the *Soja* population structure, more semi-wild lines from wide geographical locations and their sequence data need to be sampled or generated in future.

### The hybridization origin of the semi-wild soybean

The hybridization origin of the semi-wild soybean has been proposed [13], [60]. However, direct evidence in support of this hypothesis is lacking, although hybridization events between cultivated and wild soybean have been observed in natural field conditions [6], [18], [19]. In this study, several genomic evidences favor the above hypothesis.

First, the semi-wild soybeans were not grouped into an independent branch in the *Soja* phylogenetic tree (Fig. 3a). Instead, they were scattered into the wild soybean subgroup. As mentioned above, our results failed to support an independent speciation and domestication of an intermediate evolutionary type between *G. soja* and *G. max* [12]. Second, the semi-wild soybean hosts both cultivated and wild soybean novel sequences. Based on the available genomic sequences of the wild lines including Lanxi 1 and cultivars [27], [46], we identified 3.07/0.25 Mb cultivated/wild soybean-specific sequences and 3.35/0.82 Mb cultivated/wild soybean-common sequences (for detailed definition see Methods section). Using the deeply sequenced Maliaodou as an example, more than 99% of 3.07 Mb cultivated-specific sequences were covered by the reads from the Maliaodou genome (Table S9). Meanwhile, most (>95%) of the 0.82 Mb wild-common sequences and part (>12%) of the 0.25 Mb wild-specific sequences were also covered by the Maliaodou genome. Again, these findings strongly support the above hypothesis that Maliaodou originated from the hybridization of the wild and domesticated soybean. Third, unexpectedly high heterozygous rates were observed in Maliaodou and three other semi-wild lines. Soybeans are predominantly self-pollinating and usually have low outcrossing rates of <3.0% in cultivated lines but relatively higher rates (2.4–13%) in wild lines [19], [61]. This is the first time that the high heterozygous rate was observed and reported in the semi-wild soybean. Regarding the significantly higher heterozygous rate of the Maliaodou genome as compared with the 19 wild lines (*t*-test *P* value<0.1×10^−3^ for SNP and *P* = 0.4–0.9×10^−3^ for indel), a reasonable explanation is that the semi-wild line may have a recent hybrid origin rather than an intermediate evolutionary type between *G. soja* and *G. max*.

In addition, the above hypothesis is also supported by chloroplast genome data. Xu *et al*. [17] investigated chloroplast DNA SSR in 326 wild and cultivated soybean accessions and identified 52 haplotypes. However, no cultivar-specific haplotypes were found and thus hybrid swarms between cultivated and wild forms were suggested. In this study, we collected green soybean leaves of Lanxi 1 for genome re-sequencing. This generated chloroplast genomic sequences and provided us with a wild soybean chloroplast genome (Accession number: KC779227) for comparison study. Using this and the known *G. max* chloroplast genome (NC_007942) as references, the wild and cultivated lines with available genomic data [27], [46] were genotyped. No haplotype is shared by all cultivars, all wild lines or all semi-wild lines (i.e. within each group of material) used in this study (Table S10). Our results are similar to the findings by Xu and co-workers [17].

Taken together, we believe that introgressions from local wild soybeans to cultivated soybeans occurred frequently during the long domestication process of soybean, i.e. a kind of hybridization origin, which creates a complex genetic background for species in subgenus *Soja*. Several important crops are polyploidy and have originated from the hybridization of two or three ancient species, such as wheat, cotton and tobacco [62]. However, the hybridization origin of diploid crops from a domesticated line and its progenitor is rare, with only one case with genomic evidence coming from *indica* rice (*Oryza sativa*), which originated from a cross between the cultivated *japonica* rice and local wild rice [59]. For the semi-wild soybean, this should be another case of genomic evidence for the hybridization origin of diploid crops from cultivated and wild lines.

The extremely high heterozygous rates observed in Maliaodou and other semi-wild soybeans (Table S4b) suggest that their hybridization events occurred recently. This is in agreement with the fact that no record of “Maliaodou” could be found in the Chinese ancient literatures until last century (Hu & Tian 1993), although soybeans became one of the most important crops in China several thousand years ago. Wang *et al*. [18] also proposed a short creation history of semi-wild soybean based on their observation of some newly collected semi-wild lines. Additionally, the ancestry inference based on STRUCTURE result for Maliaodou indicated it is different from that of other semi-wild accessions. Based on the SNP calling result, we observed an unexpectedly high heterozygosity rate in the Maliaodou (∼50%), which indicated that this semi-wild line might originate from a recent hybridization between cultivated and wild soybeans. Tracing the location in which the Maliaodou was collected, we found that many wild soybeans are also grown in the same field, and thus genetic introgressions from wild lines to Maliaodou probably frequently occurred.

### Footprints of artificial selection in the soybean genome

Both natural and domestication selections target genes/loci controlling adaptive or agronomic traits and leave footprints of selection in the soybean genome. Several candidate domestication regions have been identified by recent genomic investigations of wild and cultivated soybeans [25], [26], [28]. Of the 282 genes located in the putative selective regions in this study (Table S7), at least 63 were also identified as putative selective genes by Li *et al*. [25] or Chung *et al*. [28] (Table S7). The gene coding for ABSCISIC ACID-INSENSITIVE 5-like protein 3 (Glyma13g03880) was detected by both of their studies as well as our study. It is one of the seed color related genes based on QTL mapping [63], and it suggests that this gene was targeted by the recent genetic improvement. Besides seed size, seed color is also a main target trait of domestication and subsequent improvement. In our study, a relatively strict criteria (*Z(H_p_)*<–4) was applied, which may exclude some putative artificially selected genes. Moreover, as shown above, 14,157 large regions (>1 kb), or about 2.8% of the reference genome of cultivated Williams 82, were not mapped by any reads from the deep sequenced (∼41×) semi-wild line Maliaodou (Table S5). These regions might be kept only in the cultivated soybean population during improvement. Taken together, the above-identified genes in the selective and un-mapping regions in this study that may relate to the artificial selection and important agronomic traits provide some candidate targets for further functional investigation.

## Supporting Information

Figure S1 **A neighbor-joining phylogenetic tree of wild (blue), semi-wild (red) and cultivated soybeans (green) labelled with seed weight per 100 seeds.**
(TIF)

Table S1 **Detailed information of the Chinese accessions of subgenus** ***Soja*** **used in this study.**
(DOC)

Table S2 **Experimental validation results for the genomic variations and PCR primers used in this study.**
(DOC)

Table S3 **Global statistics of the wild soybean Lanxi 1 and semi-wild soybean genomes.**
(XLSX)

Table S4 **Detailed information of genomic variations in the all soybean lines in this study** **relative to the reference genome Williams 82.**
(XLS)

Table S5 **Genomic structural variations between the semi-wild Maliaodou and domesticated reference genome Williams 82.**
(XLS)

Table S6 **Inference of best** ***K*** **for separating soybean subgroups using the delta** ***K*** **method.**
(DOC)

Table S7 **Position information for selective loci and genes in the selective loci in cultivated soybeans.**
(XLS)

Table S8 **GO enrichment analysis of the genes in selective regions of cultivated soybeans.**
(XLS)

Table S9 **Genomic coverage of the semi-wild soybean Maliaodou in the cultivated and wild-specific sequences of soybean.**
(DOC)

Table S10 **Chloroplast SNPs of cultivated, wild and semi-wild soybeans relative to the reference chloroplast genome (Accession no. NC_007942).**
(XLS)
