# Supplementary figures and images for: Genome Re-Sequencing of Semi-Wild Soybean Reveals a Complex Soja Population Structure and Deep Introgression
Source: PLoS One. 2014 Sep 29;9(9):e108479. doi: 10.1371/journal.pone.0108479 (PMC4181298; doi:10.1371/journal.pone.0108479)

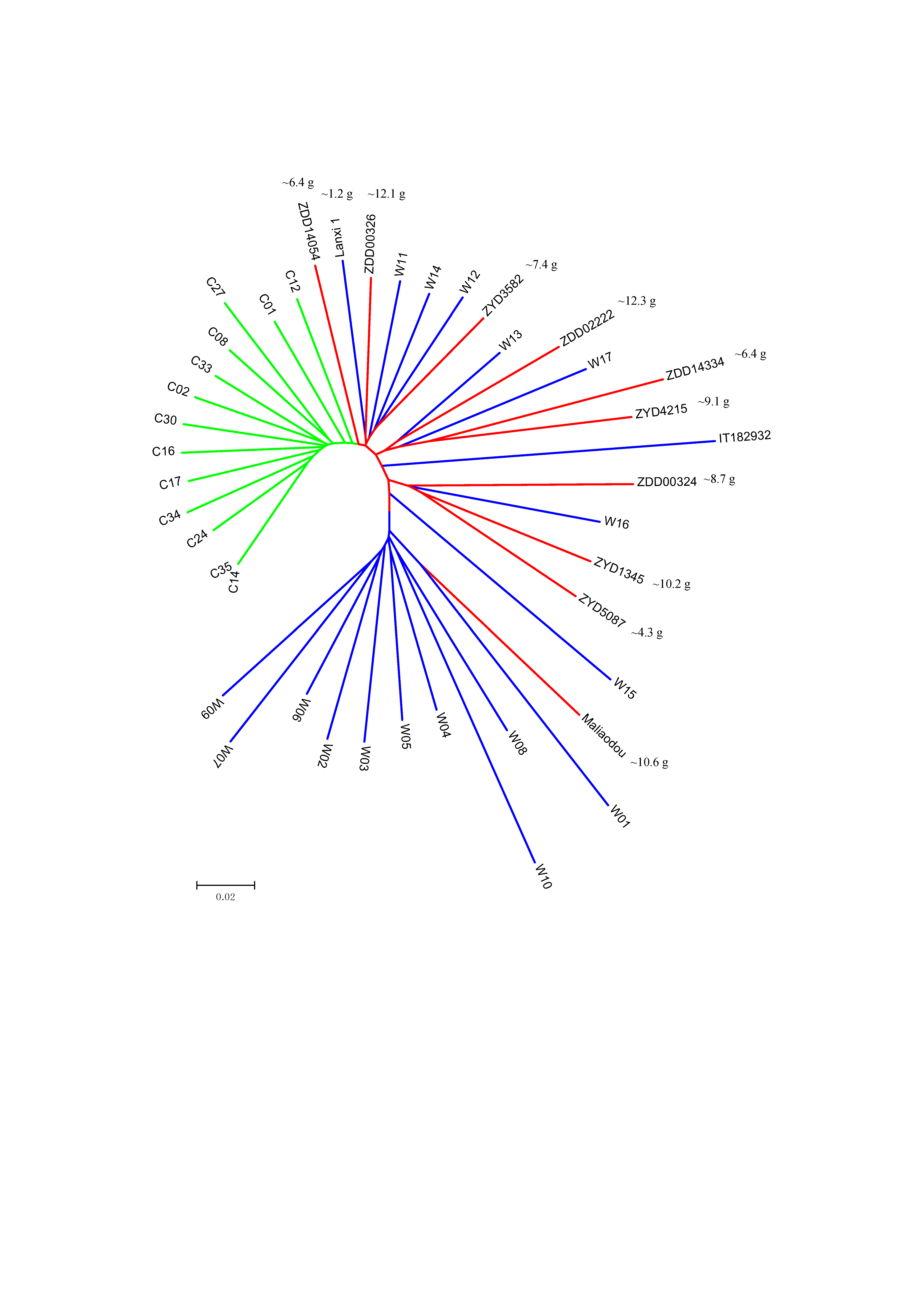

Supplement: Figure S1 — A neighbor-joining phylogenetic tree of wild (blue), semi-wild (red) and cultivated soybeans (green) labelled with seed weight per 100 seeds. (TIF) [file pone.0108479.s001.tif]
