# Supplementary material for: Genome Re-Sequencing of Semi-Wild Soybean Reveals a Complex Soja Population Structure and Deep Introgression
Source: PLoS One. 2014 Sep 29;9(9):e108479. doi: 10.1371/journal.pone.0108479 (PMC4181298; doi:10.1371/journal.pone.0108479)
Supplement: Table S1 — Detailed information of the Chinese accessions of subgenus Soja used in this study. (DOC) [file pone.0108479.s002.doc]

**Table S1** Detailed information of Chinese accessions of subgenus *Soja* used in this study

| Species | Accession no. | Weight (g) per 100 seeds | Origin |
| --- | --- | --- | --- |
| Semi-wild | ZDD00324 | 8.67 | Institute of Crop Sciences, CAAS |
|  | ZDD00326 | 12.06 | Institute of Crop Sciences, CAAS |
|  | ZDD14054 | 12.04 | Institute of Crop Sciences, CAAS |
|  | ZDD14334 | 6.41 | Institute of Crop Sciences, CAAS |
|  | ZDD02222 | 12.32 | Institute of Crop Sciences, CAAS |
|  | ZYD 1345 | 10.15 | Institute of Crop Sciences, CAAS |
|  | ZYD 3582 | 7.44 | Institute of Crop Sciences, CAAS |
|  | ZYD 4215 | 9.11 | Institute of Crop Sciences, CAAS |
|  | ZYD5087 | 4.34 | Institute of Crop Sciences, CAAS |
|  | Maliaodou | 10.55 | Institute of Crop Sciences, Zhejiang University |
| Wild | Lanxi1 | 1.22 | Institute of Crop Sciences, Zhejiang University |
