# Supplementary material for: Genome Re-Sequencing of Semi-Wild Soybean Reveals a Complex Soja Population Structure and Deep Introgression
Source: PLoS One. 2014 Sep 29;9(9):e108479. doi: 10.1371/journal.pone.0108479 (PMC4181298; doi:10.1371/journal.pone.0108479)
Supplement: Table S2 — Experimental validation results for the genomic variations and PCR primers used in this study. (DOC) [file pone.0108479.s003.doc]

**Table S2** Experimental validation results for the genomic variations and PCR primers used in this study.

| Chromosome | Genomic position of variations | Forward primer(5'-3') | Revrse primer(5'-3') | Variation type | Validation results* |
| --- | --- | --- | --- | --- | --- |
| Gm01 | 6861 | GTTATGCCAAGCAAGCCAACA | CCTGGTTGTCGAAAGAACAATC | SNP | ND |
| Gm01 | 23629 | GTTTCGGAGCCATGAGAACA | AGTACCCCTTCTGCAAACGA | SNP | YES |
| Gm01 | 24939 | CCCACATCTTTGGCTCAATC | GGGCTTTGGCCTTTAACTTC | SNP | YES |
| Gm01 | 28368 | GGAAATTGGCATTGGCATTA | CAAAGGCAGAACTATTGAGCA | InDel | YES |
| Gm01 | 34533 | ATGAGCCTGATTCTGGATGG | GTATGGAGGGTTGGTTGTGG | InDel | YES |
| Gm01 | 51636 | CAAGCAAAGCACATCAACCT | GGTGTAGATGATGCACATGGT | InDel | ND |
| Gm01 | 71421 | AACATTGCTCCATTGCACAC | GAAGTCCTCTAACACCTGATACG | InDel | YES |
| Gm02 | 16205 | GGGATTAGCTCTGAGTGATGCT | TTGATTTACGCTGCACCTCA | InDel | YES |
| Gm02 | 39994 | GCTTCTGATTTGACGTGCAT | GGATTCCAGCTTTTTCAATCC | InDel | YES |
| Gm02 | 81983 | GCACACCAAACAACACTTGG | CCCTGAGGCTCTGATACCTG | InDel | YES |
| Gm02 | 87905 | GCATGAAAGGTGCTTGTGTG | CACGACCGTCATGAAATGAG | InDel | YES |
| Gm02 | 97804 | GAAGTTTCCCAAGAGCGAGA | GCAGGAGTGCATGACCTTTT | InDel | YES |
| Gm02 | 109198 | GCCGAATAGTTGTGCGGTAT | GCTTGTCGCAGTAAGGAAGG | InDel | YES |
| Gm02 | 109515 | CCTTCCTTACTGCGACAAGC | AGAGCCAGCATCGTTGAGAT | SNP | YES |
| Gm02 | 110228 | GGTCGGCATGTTAGGTGAGA | GTGTTCAGCTCATGATCCGTG | SNP | YES |
| Gm02 | 134919 | TACCTGCGCCCAATTCAACT | AGACATCCAGCCGCCATAAT | SNP | YES |
| Gm02 | 140730 | GTCGTGTTCCCGATATTGCT | GCGAATCCACTTTCAGAAGC | InDel | ND |
| Gm02 | 163488 | CCTTGGGAACAAAATCATTG | TGAGAATTGTCGAACGTGGT | InDel | YES |
| Gm02 | 165356 | GTCGGCCGTAAAAACTTCAG | GATGGATCAATTCGGTAGGG | SNP | YES |
| Gm02 | 300003 | ACAGAGGCATGAATACCAAGGG | CCATCTTTGCACATGAGGATTGG | InDel | YES |
| Gm02 | 307043 | ATTGGGCTCAATTGTGAAGC | TCCCCACAAAGTGATCTAGC | InDel | YES |
| Gm03 | 28333 | AACCGGATTAGGTTGTGCTG | ATGTCCATAAGGCCCATGAT | InDel | YES |
| Gm03 | 701481 | TGGGAAAGCTTAAGGGTGTG | ATACCCATTTCCACCCAACA | InDel | ND |
| Gm03 | 710195 | TGTGGATCCTCTTATGAAGGTG | GCAATGTGAAAGGGACCACT | SNP | YES |
| Gm03 | 719272 | TGAACTTGCGAATTGCTCAC | TTGGCAGCGACTAAGTTTCA | SNP | YES |

*ND: not determined yet
