# Supplementary material for: Genome Re-Sequencing of Semi-Wild Soybean Reveals a Complex Soja Population Structure and Deep Introgression
Source: PLoS One. 2014 Sep 29;9(9):e108479. doi: 10.1371/journal.pone.0108479 (PMC4181298; doi:10.1371/journal.pone.0108479)
Supplement: Table S6 — Inference of best K for separating soybean subgroups using the delta K method. (DOC) [file pone.0108479.s007.doc]

**Table S6** Inference of best *K* for separating soybean subgroups using the delta *K* method

| *K* | L(K) | Stdev | L'(*K*) | L''(*K*) | [L''*K*] | Delta *K* |
| --- | --- | --- | --- | --- | --- | --- |
| 2 | -147590.80 | 161.50 |  |  |  |  |
| 3 | -139282.35 | 131.80 | 8308.45 | 1882.01 | 1882.01 | 14.28 |
| 4 | -132855.91 | 391.93 | 6426.44 | 281.72 | 281.72 | 0.72 |
| 5 | -126711.20 | 712.30 | 6144.71 | 2242.75 | 2242.75 | 3.15 |
| 6 | -122809.24 | 1063.14 | 3901.96 | 297.23 | 297.23 | 0.28 |
| 7 | -119204.50 | 3417.54 | 3604.74 | -2409.60 | 2409.60 | 0.71 |
| 8 | -113190.16 | 2025.18 | 6014.34 | 5116.35 | 5116.35 | 2.53 |
| 9 | -112292.18 | 3379.95 | 897.99 | 897.99 | 897.99 | 0.27 |
