# Supplementary material for: Genome Re-Sequencing of Semi-Wild Soybean Reveals a Complex Soja Population Structure and Deep Introgression
Source: PLoS One. 2014 Sep 29;9(9):e108479. doi: 10.1371/journal.pone.0108479 (PMC4181298; doi:10.1371/journal.pone.0108479)
Supplement: Table S9 — Genomic coverage of the semi-wild soybean Maliaodou in the cultivated and wild-specific sequences of soybean. (DOC) [file pone.0108479.s010.doc]

**Table S9.** Genomic coverage of the semi-wild soybean Maliaodou in the cultivated and wild-specific sequences of soybean.

| Sequences | Genomic sequence (Mb) | Coverage (%) | Reference genome |
| --- | --- | --- | --- |
| Cultivar-specific | 3.07 | 99.5 | Williams 82 |
| Cultivar common | 3.35 | 91.2 | Williams 82 |
| Wild-specific | 0.25 | 12.1 | Lanxi 1 |
| Wild common | 0.82 | 95.4 | Lanxi 1 |
